# Cryomicrobial Ecology: Still Much To Learn about Life Left Out in the Cold

**DOI:** 10.1128/mSystems.00852-21

**Published:** 2021-09-07

**Authors:** Jackie Goordial

**Affiliations:** a University of Guelph, Guelph, Ontario, Canada

**Keywords:** astrobiology, cryomicrobiology, extremophile, permafrost, polar, psychrophile

## Abstract

Studies from cryoenvironments on Earth have demonstrated that microbial life is widespread and have identified microorganisms that are metabolically active and can replicate at subzero temperatures if liquid water is present. However, cryophiles (subzero-growing organisms) often exist in low densities in the environment and their growth rate is low, making them difficult to study. Compounding this, a large number of dormant and dead cells are preserved in frozen settings. Using integrated genomic and activity-based approaches is essential to understanding the cold limits of life on Earth, as well as how cryophilic microorganisms are poised to adapt and metabolize in warming settings, such as in thawing permafrost. An increased understanding of cryophilic lifestyles on Earth will also help inform how (and where) we look for potential microbial life on cold planetary bodies in our solar system such as Mars, Europa, and Enceladus.

## COMMENTARY

Cryoenvironments exist either continuously or predominately at subzero temperatures. They occur in polar and alpine regions and include visually stunning large-scale features such as glaciers, sea ice, permanently ice-covered lakes, and polygonal terrain associated with permafrost but can also extend into the atmosphere where cold temperatures dominate in clouds and dust particles. Studies from both cryoenvironments and laboratories on Earth have clearly demonstrated that microbial life is widespread in cold environments, can be metabolically active, and can replicate at low, subzero temperatures if sufficient liquid water is present, even if present only in thin films (1, 2). As ice forms at subzero temperatures, liquid water becomes unavailable to microbial life. Due to the distinct stressor that temperature-related low water activity imposes on a cell, a distinction is made between psychrophilic organisms (organisms which have temperature optima at cold temperatures) and cryophiles (organisms capable of growth below freezing temperatures). The ability to replicate at subzero temperatures has been demonstrated in all three domains of life (1). The current record for bacterial cellular division at low temperature is −15°C by Planococcus halocryophilus OR1, isolated from Arctic active-layer soils (the top layer above permafrost that thaws seasonally in the summer) (2). The fungal record is held by a *Rhodotorula* yeast, reported from spoiled, frozen peas at −18°C (3), the relatively mundane habitat hinting that cryophilic (or at minimum psychrotolerant) microorganisms may be more abundant and ubiquitous than previously appreciated in our daily lives. These examples and most of what is known about subzero growth and the adaptive traits required to survive in cold environments are from isolated organisms which can be cultivated in the laboratory. In the laboratory, cryophilic organisms are typically fastidious and require patience due to long doubling times at subzero temperatures (weeks to months), a result of generally lower metabolic and kinetic rates at low temperatures. As a result, there are a limited number of characterized cryophilic organisms and only a few available genomes (to my best knowledge, fewer than 20 available genomes at the time of writing are from confirmed subzero-growing organisms). As the vast majority of microorganisms cannot yet be cultivated, the identity, metabolic functions, and mechanisms for cold adaptation of environmental cryophilic organisms remain poorly understood. More research is needed to reveal how microbial activity and dormancy in cryoenvironments relate to diversity, evolution, and biogeochemical cycling on Earth, and potentially beyond in the field known as astrobiology. To tackle these unknowns, our research employs a variety of *in situ* (field) and laboratory approaches, combining molecular sequencing with microbial physiology and activity assays.

## CRYOPHILES IN A CHANGING CLIMATE

A comprehensive understanding of how microbial diversity, activity, adaptation, and dormancy affect biogeochemical cycling in cryoenvironments is increasingly relevant in the face of climate change. For example, microbial communities in thawing permafrost are making the shift from dormancy to activity, with the potential to process vast amounts of carbon, and with the metabolic capacity for pathogenicity (4) and antibiotic resistance already shown (5). Permafrost contains the world’s largest reservoir of C and accounts for 34% of the world’s coasts (6, 7). Warming occurring in polar regions can accelerate the microbial degradation of stored C, resulting in the release of greenhouse gases (GHGs) such as CO_2_, CH_4_, and N_2_O. This feedback can accelerate climate change, but the magnitude and timing of GHG emissions and their impact on climate change remain uncertain (8). Permafrost soils do not need to thaw into an active layer to result in GHG release; only a few degrees centigrade of warming can result in methane emissions (9). Measuring the quantities and fate of degrading permafrost carbon, and on what time scale it is processed terrestrially and in nearshore environments, is crucial to understanding how carbon flux (GHG emissions) affected climate change in the past and how it will impact it in the future. Microorganisms are key drivers of greenhouse gas flux and thus a critical piece to understand, with an emphasis on activity and physiology. Not all permafrost is equal, and our current research strives to incorporate geology, chemistry, and the indigenous microbial communities from permafrost section type as context for evaluating the potential for increased activity and/or adaptation. Our group foresees that an improved understanding of cryomicrobial ecology will inform current and future predictions of biogeochemical cycling in polar settings poised for rapid change.

## CRYOPHILIC LIFE—SLOW AND STEADY?

There is evidence that in many environmental settings, the majority of microorganisms are dormant or in a (viable) state of nongrowth (10), and thus, bulk genomic analyses do not represent a true picture of active *in situ* microbial processes; this may be especially true in settings akin to a laboratory freezer, where DNA from dead and dormant microorganisms can be preserved on long time scales, emphasizing the critical need to incorporate physiology and activity measurements into our understanding of subzero settings. Though some permafrost communities may be dominated by dormant microorganisms (11), dormancy may not be the best strategy for a microorganism to avoid cellular damage when frozen “in place” on long time scales, and rather organisms that are able to carry out essential functions such as DNA and protein repair in a state of “maintenance” may hold an advantage in these settings (12, 13). Indeed, in some permafrost soils there is evidence that even cells capable of forming spores are not necessarily dormant *in situ* (14). Previously, my work paradoxically isolated non-spore-forming cryophilic isolates from dry permafrost soils thought to be otherwise devoid of active life (15–17). Our current work now investigates whether such cold-active microorganisms may be persisting in such environments cryptically, with metabolic rates too low and metabolism too slow to detect using standard microbiological approaches. To do this, our current work takes advantage of single-cell microbiology flow cytometry sorting techniques that can sequence the genomes of individual cells, even in low-biomass environments (18, 19). When combined with fluorescent tagging techniques for active and replicating cells (20), cell sorting permits analysis of the genomics and activity of cryophilic microbes without requiring a cultivated isolate. Individual cells with damaged membranes, active oxidoreductases, or that are actively translating new proteins can be distinguished within communities, allowing us to quantify and examine the metabolic potential underlying these different metabolic states in the environment under changing and static conditions. I envision these experiments will inform our fundamental understanding of the cold limits of life, as well as aid our understanding of how future communities will assemble and adapt (since evolution requires replication) to a warming world.

## THE PSYCHROPHILE NEXT DOOR

Cryophilic organisms are active in environments most of us would not consider to be extreme, such as the location of my institution in Guelph, Ontario (Canada), which experiences subzero temperatures for several months of the year. Microbial activity occurs throughout the winter in frozen agricultural soils and forest soils (21, 22), with microorganisms seemingly resistant to multiple freeze-thaw cycles. Additionally, we may encounter cryophiles that cause food spoilage in frozen foods (3). Enzymes from cold-adapted microorganisms are used in “cold-water” laundry detergents, and ice-nucleating proteins from bacteria are used in snow machines to ensure skiers have ample snow even when conditions are not ideal. Through our work, and informed by activity-based ‘omic approaches, we are building a library of isolated cryophiles, with rich potential for biotechnology and applied environmental microbiology. We will investigate this potential with our collection of organisms for applications such as winter soil management practices, as well as making this collection generally available for other researchers to use.

## THE PSYCHROPHILE ON THE NEXT-DOOR … PLANETARY BODY?

**FIG 1:**
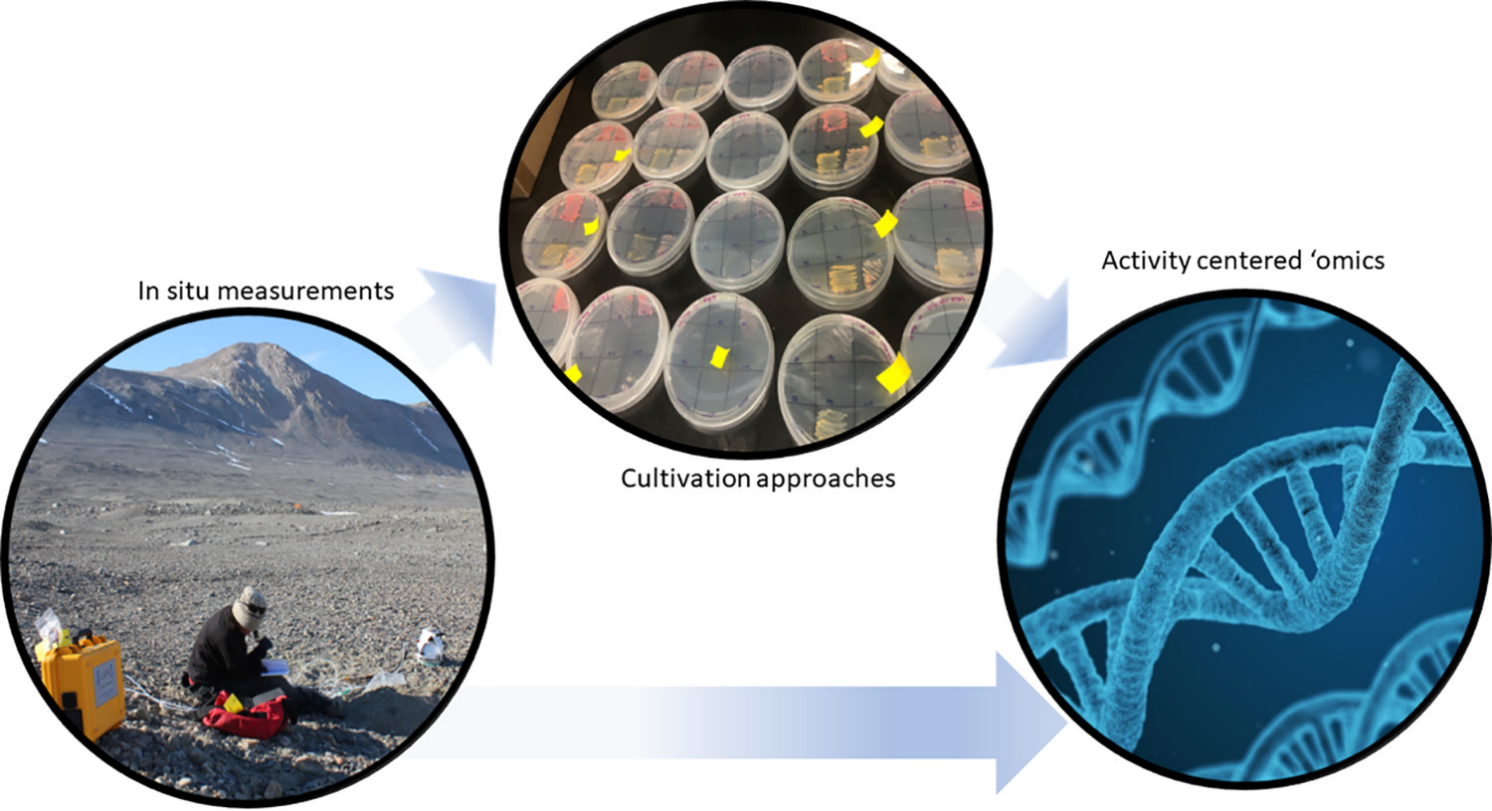
Our approach to analyzing dry permafrost includes *in situ* measurements of activity, cultivation attempts, and activity-centered ‘omics such as the use of cell sorting in conjunction with activity-based fluorescent stains.

Within our solar system, there are multiple promising planetary bodies where microbial life could potentially exist (extant life) or could have existed in the past. Cold temperatures characterize primary targets such as the planet Mars and the moons of Saturn and Jupiter, Enceladus, Titan, and Europa. Cryoenvironments are some of the best analogs to inform where, and how, we could detect life elsewhere. Our current research focuses on dry permafrost, frozen soils with negligible water content similar to those found on Mars (23) (Fig. 1). While Mars is a cold and dry planet at present, in the past it was relatively warmer and wetter with evidence of the presence of vast oceans (24). If life was once present on Mars, our work investigating dormancy and low metabolic rates in polar settings can shed light on the potential for such life to have persisted in the subsurface. Cryophile research into the limits of life can inform planetary protection concerns for eventual manned flights to Mars, as well as potentially one day play a role in environmental microbiology applications on other planetary bodies.

In summary, though cryophilic life is known to be widespread and active in the environment, fundamental questions remain and there is still much left to learn. The activity of cryophilic organisms in polar settings could have global-scale effects through GHG release and is a pressing concern to understand. Our research hopes to improve understanding of cryomicrobial ecology, to better predict microbial response under changing conditions, to understand the limits of life on Earth and beyond, and to tap into significant biotechnological potential.
